# Author Correction: Pharmacogenomic screening identifies and repurposes leucovorin and dyclonine as pro-oligodendrogenic compounds in brain repair

**DOI:** 10.1038/s41467-025-55864-4

**Published:** 2025-01-09

**Authors:** Jean-Baptiste Huré, Louis Foucault, Litsa Maria Ghayad, Corentine Marie, Nicolas Vachoud, Lucas Baudouin, Rihab Azmani, Natalija Ivljanin, Alvaro Arevalo-Nuevo, Morgane Pigache, Lamia Bouslama-Oueghlani, Julie-Anne Chemelle, Marie-Aimée Dronne, Raphaël Terreux, Bassem Hassan, François Gueyffier, Olivier Raineteau, Carlos Parras

**Affiliations:** 1 Paris Brain Institute, Sorbonne Université, Inserm U1127, CNRS UMR 7225, Hôpital Pitié-Salpêtrière, Paris, France; 2 Univ Lyon, Université Claude Bernard Lyon1, Inserm, Stem Cell and Brain Research Institute U1208, Bron, France; 3 Équipe ECMO, Laboratoire de Biologie Tissulaire et d’Ingénierie (LBTI), UMR5305, Lyon, France; 4 Claude Bernard University, UMR5558 Laboratoire de Biométrie et Biologie Evolutive, CNRS, Villeurbanne, France

**Keywords:** Virtual screening, Neonatal brain damage, Multiple sclerosis

Correction to: *Nature Communications* 10.1038/s41467-024-54003-9, published online 13 November 2024

In this article the author’s name Natalija Ivljanin was incorrectly written as Natalija Ivjanin. The original article has been updated.

