# Supplementary material for: Pharmacogenomic screening identifies and repurposes leucovorin and dyclonine as pro-oligodendrogenic compounds in brain repair
Source: Nat Commun. 2024 Nov 13;15:9837. doi: 10.1038/s41467-024-54003-9 (PMC11561360; doi:10.1038/s41467-024-54003-9)
Supplement: Supplementary file 1 — Supplementary Information [file 41467_2024_54003_MOESM1_ESM.pdf]

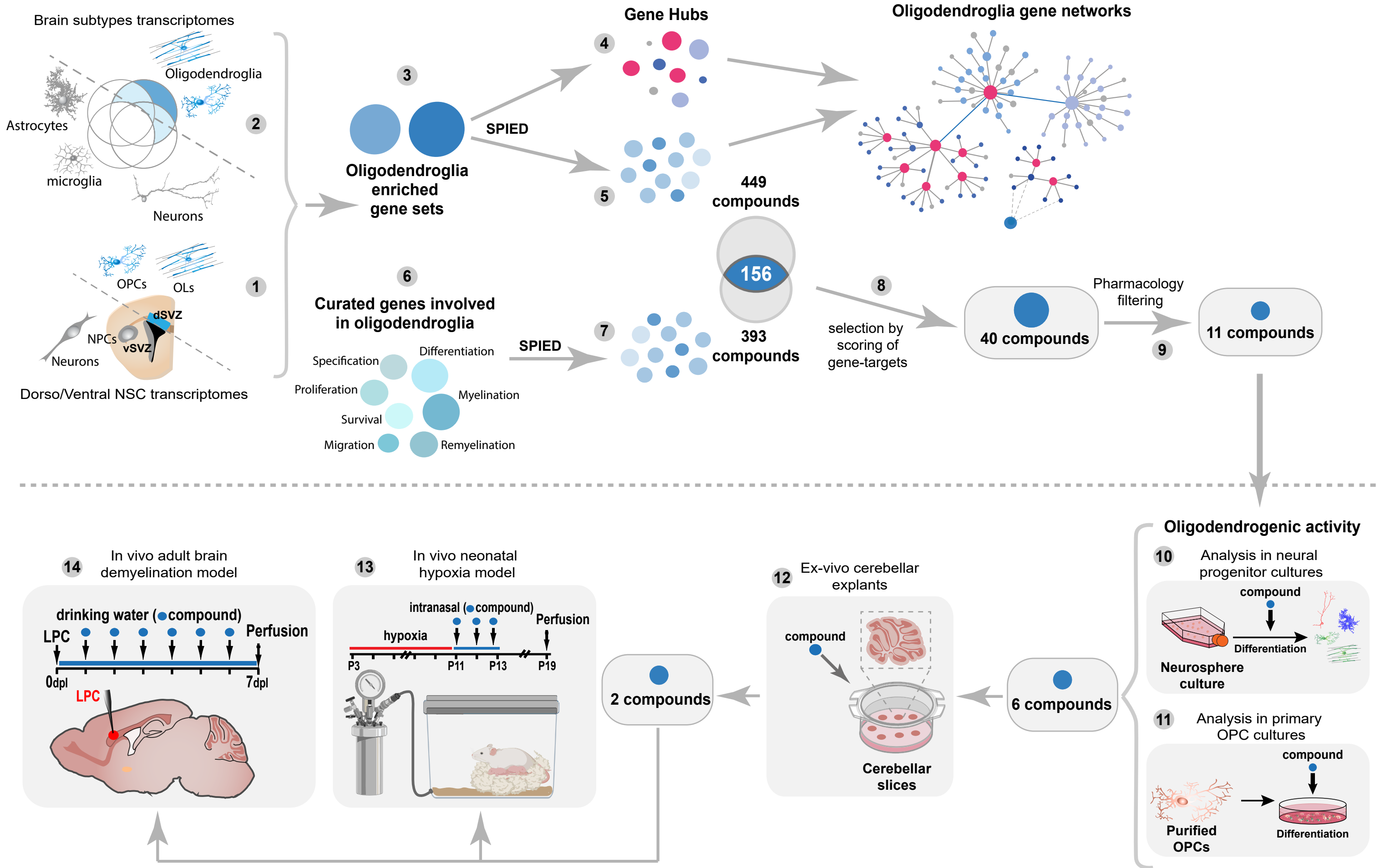
Brain subtypes transcriptomes
Oligodendroglia
Astrocytes
microglia
Neurons
2
OPCs
OLs
1
dSVZ
NPCs
vSVZ
Neurons
Dorso/Ventral NSC transcriptomes
3
Oligodendroglia enriched gene sets
SPIED
Gene Hubs
4
5
449 compounds
Oligodendroglia gene networks
156
393 compounds
6
Curated genes involved in oligodendroglia
Specification
Differentiation
Proliferation
Myelination
Survival
Migration
Remyelination
SPIED
7
8
selection by scoring of gene-targets
40 compounds
Pharmacology filtering
9
11 compounds
Oligodendrogenic activity
10
Analysis in neural progenitor cultures
compound
Differentiation
Neurosphere culture
11
Analysis in primary OPC cultures
compound
Purified OPCs
Differentiation
6 compounds
12
Ex-vivo cerebellar explants
compound
Cerebellar slices
2 compounds
13
In vivo neonatal hypoxia model
intranasal (compound)
hypoxia
Perfusion
P3
P11
P13
P19
14
In vivo adult brain demyelination model
drinking water (compound)
LPC
Perfusion
0dpl
7dpl
LPC

Supplementary figure 1. Summary scheme of the screening strategy and compound selection process. Schematics representing the in-silico (top) and wet-lab (bottom) strategies. In-silico bioinformatic strategy allowed the generation of a large oligodendroglial transcriptional signature (steps 1-3) and gene hubs of the oligodendroglial gene networks (step 4) used to generate a list of compounds with putative pro-oligodendrogenic and (re)myelinating activities (step 5). Compounds were ranked by their large pro-oligodendrogenic activities through expert curation of their regulated target genes (steps 6-8), and then by their pharmacological properties (step 9), with a final selection of the top 11 compounds. Wet-lab strategy to assess the pro-oligodendrogenic activity of the selected compounds in the culture of neural progenitor cells (step 10) and OPCs (step 11), resulting in 6 compounds being selected for further activity assessment in *ex vivo* cerebellar explant cultures (step 12). Finally, the top two compounds were used *in vivo* in a model of preterm birth injury (neonatal hypoxia, step 13) and a model of adult de/remyelination (step 14). Numbers in grey circles indicate the different steps of the project as detailed in the main text.

OligoScore

https://oligoscore.icm-institute.org/

Oligodendrogenesis curated gene list

Liste of genes involved in oligodendrogenesis (from the literature)

- Which process (Prolif, Diff...)?
- What impact (1, 2 or 3)?
- Which effect (Positive or Negative)?

>1000 publications

>430 genes

	Specif	Prolif	Migrat	Survival	Differ	Myelin	Remyel
Olig2	3	2	0	0	2	2	1
Olig1	1	-1	0	0	2	2	2
Ascl1	2	1	0	0	2	0	1
Pdgfra	1	3	0	0	0	0	0
Mbp	1	0	0	0	1	2	0
Gpr17	1	0	0	-1	2	-1	-1
...							
**Total**	**29**	**75**	**42**	**21**	**162**	**221**	**50**

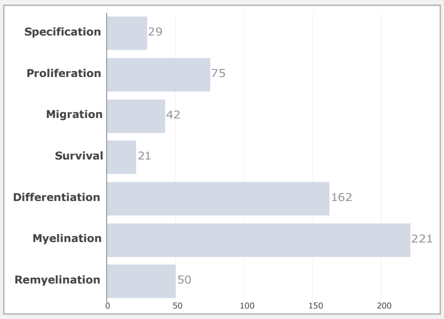

1- To assess the implication of genes in different processes of oligodendrogenesis

Gene list (Selection)

Aatk	Enpp2	Mal	Serinc5
Acox1	Enpp6	Mapk3	Sirt2
Acss2	Ermn	Marcks	Sox10
Adamts4	Fa2h	Mbp	Sox8
Aplp2	Fabp7	Mobp	Sulf2
Apoe	Fasn	Mog	Thra
App	Fez1	Myrf	Tnr
Aspa	Fkbp1a	Ndrg1	Tns3
Bcan	Fth1	Nfasc	Tppp
Bcas1	Fyn	Nkx6-2	Tyro3
Cadm4	Gamt	Olig1	Ugt8a
Cd9	Gas7	Olig2	Unc5b
Cdc42	Gatm	Omg	Vamp2
Cdk4	Gipc1	Opalin	
Cdk5	Gjb1	Pdgfra	
Ckb	Gjc2	Plat	
Cldn11	Gnas	Plekha1	
Cmtm5	Gpr17	Pllp	
Cnp	Gpr37	Plp1	
Cntn1	Gpr62	Ppp1r16b	
Cntn2	Gpx1	Psap	
Ctnnb1	Gsn	Ptn	
Ctsd	Hdac11	Ptprz1	
Cyp51	Hexb	Qk	
Ddx5	Hmgcr	Rac1	
Dusp15	Kcnj10	Rhoa	
Ednrb	Lpar1	Rnd2	
Efnb3	Lrp1	Rras2	
Eif1b	Mag	S1pr5	

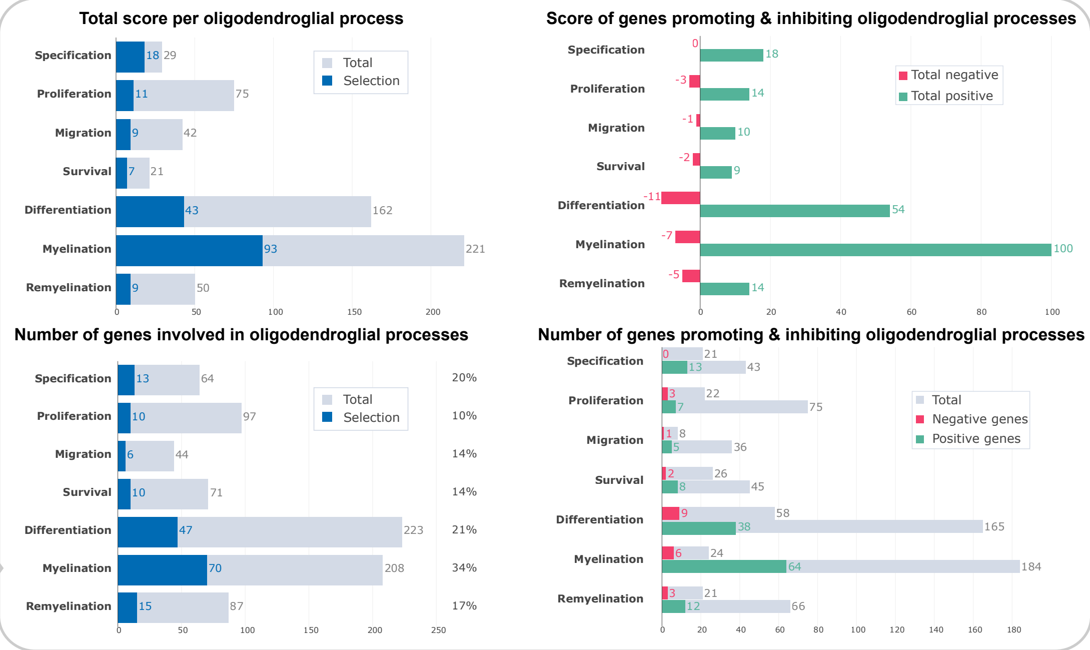

2- To evaluate the impact of a list of deregulated genes in different processes of oligodendrogenesis

Deregulated genes

LogFC Mutant vs Ctrl

Gene	LogFC
Olig2	-1,80
Olig1	-2,14
Ascl1	0,56
Pdgfra	-0,38
Mbp	-0,39
Sox2	0,87
Gpr17	-1,15

Impact of each gene in each process

Score * LogFC

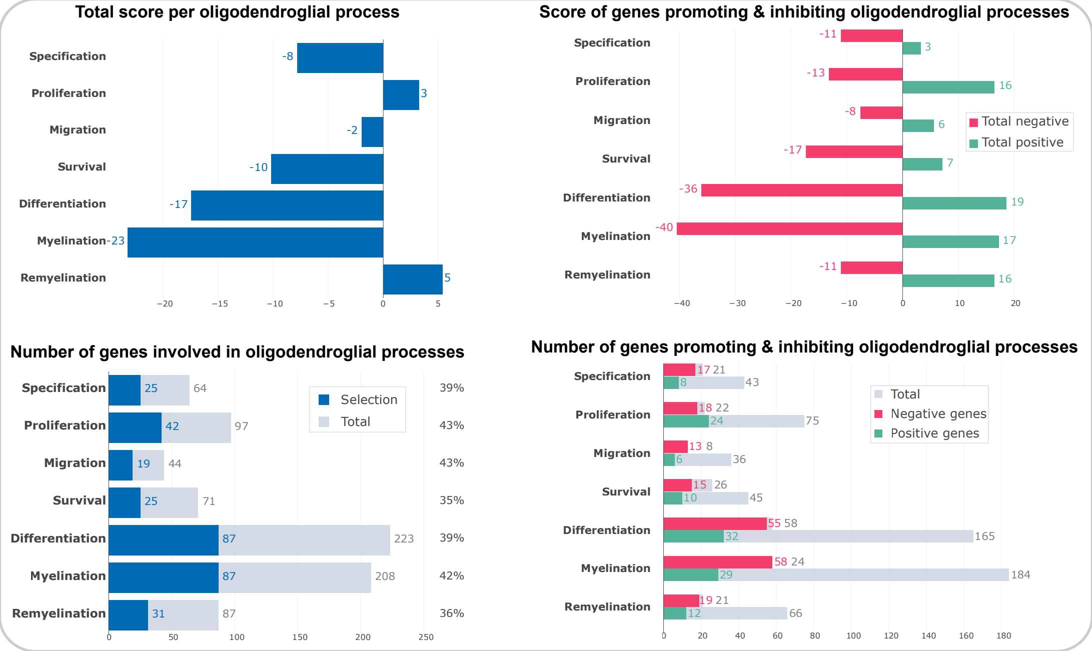

Supplementary figure 2. OligoScore, a new web resource to evaluate gene sets for their involvement in oligodendrogenesis. Schematics illustrating the OligoScore resource, a curation strategy currently encompassing over 430 genes and 1000 publications that report the functional implication (either by loss-of-function or gain-of-function experiments) of a given gene in each aspect (process) of oligodendrogenesis (i.e., specification, proliferation, migration, survival, differentiation, myelination, and remyelination). Detailed statistics obtained either from queries using either (1) a gene set (i.e., list of genes) or (2) a list of deregulated genes (i.e., genes and logarithmic fold-changes, logFC) are provided as barplots.

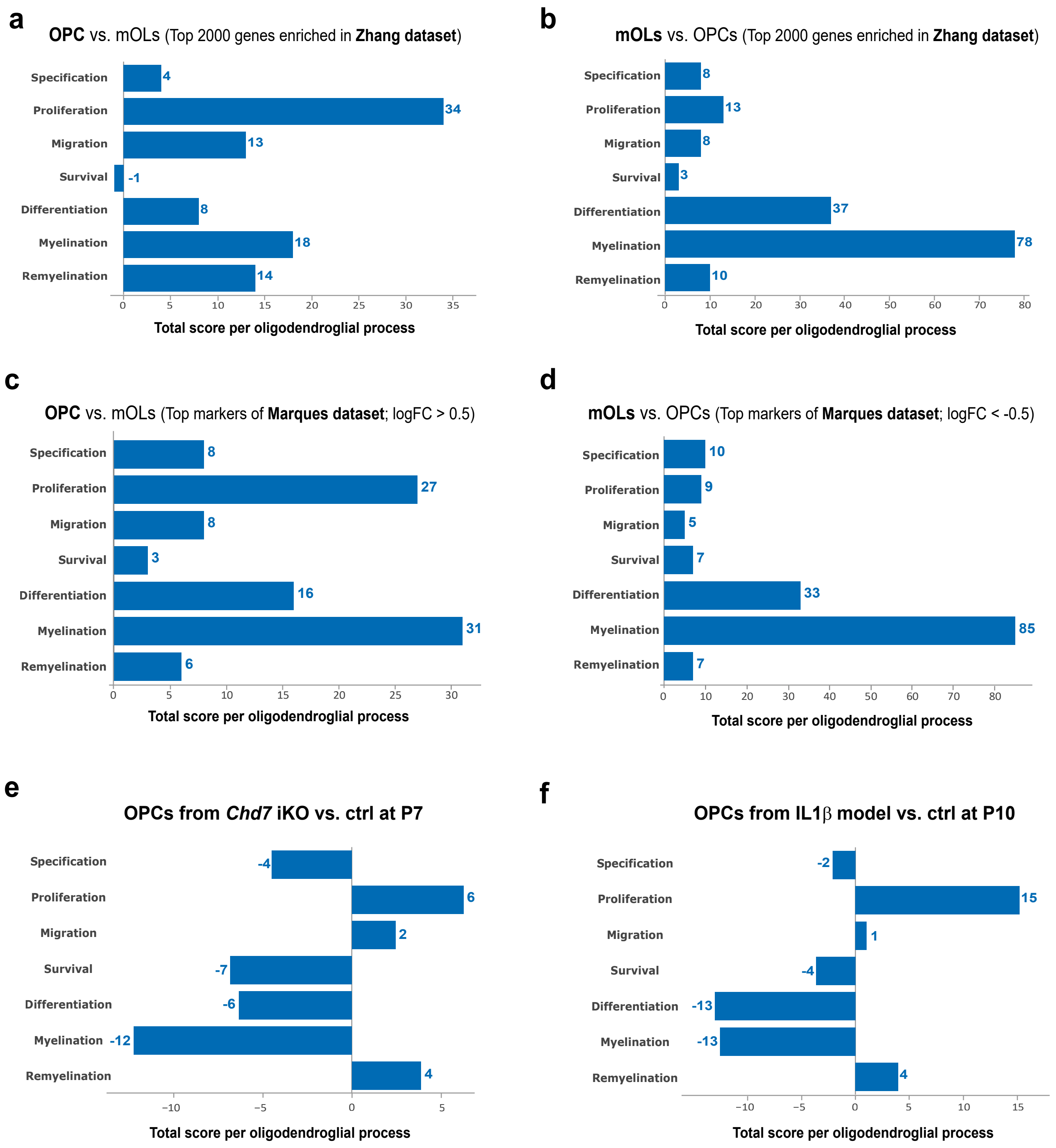
a
OPC vs. mOLs (Top 2000 genes enriched in Zhang dataset)
Specification 4
Proliferation 34
Migration 13
Survival -1
Differentiation 8
Myelination 18
Remyelination 14
0 5 10 15 20 25 30 35
Total score per oligodendroglial process
b
mOLs vs. OPCs (Top 2000 genes enriched in Zhang dataset)
Specification 8
Proliferation 13
Migration 8
Survival 3
Differentiation 37
Myelination 78
Remyelination 10
0 10 20 30 40 50 60 70 80
Total score per oligodendroglial process
c
OPC vs. mOLs (Top markers of Marques dataset; logFC > 0.5)
Specification 8
Proliferation 27
Migration 8
Survival 3
Differentiation 16
Myelination 31
Remyelination 6
0 5 10 15 20 25 30
Total score per oligodendroglial process
d
mOLs vs. OPCs (Top markers of Marques dataset; logFC < -0.5)
Specification 10
Proliferation 9
Migration 5
Survival 7
Differentiation 33
Myelination 85
Remyelination 7
0 10 20 30 40 50 60 70 80
Total score per oligodendroglial process
e
OPCs from Chd7 iKO vs. ctrl at P7
Specification -4
Proliferation 6
Migration 2
Survival -7
Differentiation -6
Myelination -12
Remyelination 4
−10 −5 0 5
Total score per oligodendroglial process
f
OPCs from IL1β model vs. ctrl at P10
Specification -2
Proliferation 15
Migration 1
Survival -4
Differentiation -13
Myelination -13
Remyelination 4
−10 −5 0 5 10 15
Total score per oligodendroglial process

Supplementary figure 3. OligoScore validation using expression and deregulated transcriptional signatures. (**a-d**) Querying of OligoScore with genes enriched in OPC vs. myelinating OLs (mOLs), and vice versa, using either (**a**) postnatal purified brain cell transcriptomes (Zhang et al., 2024) or (**c**) oligodendroglial single-cells transcriptomes (Marques et al., 2016, 2018; see also Methods and *OligoScore_validation_tables* file). In both cases, OligoScore highlights that while OPC gene programs are involved in several processes of oligodendrogenesis, including specification, proliferation, migration, differentiation, and myelination (**a, c**), mOL-enriched genes are mainly involved in differentiation and myelination (**b, d**). (**e, f**) Querying OligoScore (genes and fold-changes) with genes dysregulated in OPCs upon a genetic (postnatal day 7 OPCs with an induced knockout for *Chd7,* compared to controls; Marie et al., 2018), or an environmental (P10 OPCs in the systemic IL1b-mediated neonatal neuroinflammatory model, Schang et al., 2022) perturbation. This analysis highlights the deregulated processes identified in these studies, such as reduced survival, and differentiation of *Chd7* iKO OPCs (**e**), and increased proliferation and reduced differentiation of OPCs in the IL1b-model (**f**).

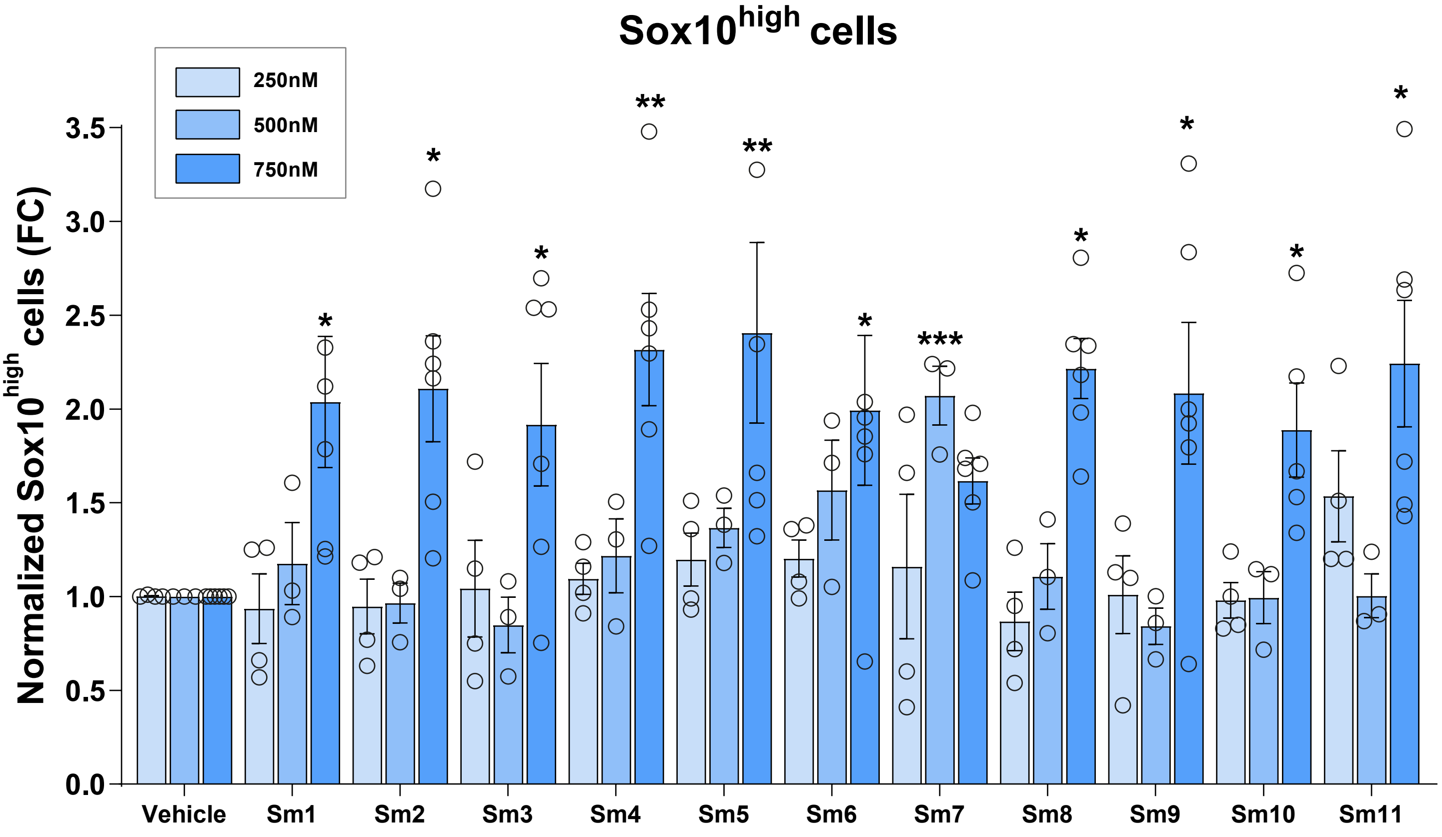

Sox10^high cells
250nM
500nM
750nM
Normalized Sox10^high cells (FC)
0.0
0.5
1.0
1.5
2.0
2.5
3.0
3.5
Vehicle
Sm1
Sm2
Sm3
Sm4
Sm5
Sm6
Sm7
Sm8
Sm9
Sm10
Sm11
*
**
***

Supplementary figure 4. Dose effect of compounds' pro-oligodendrogenic activity in neonatal neural progenitor cultures. Quantifications of $Sox10^{high}$ cells (iOLs) in cultures treated with compounds at 250nM (N=4), 500nM (N=3), and 750nM (N=6), showing the best effect at 750nM. Data are presented as mean ± SEM of fold change normalized to vehicle. Statistical analysis used linear mixed-effects models followed by Type II Wald chi-square tests. Each dot represents a biological replicate. *p < 0.05; **p < 0.01; ***p < 0.001. Exact p-values, sample sizes (represented in the dot plots), and source data are provided in the Source Data file and in Methods Table 3.

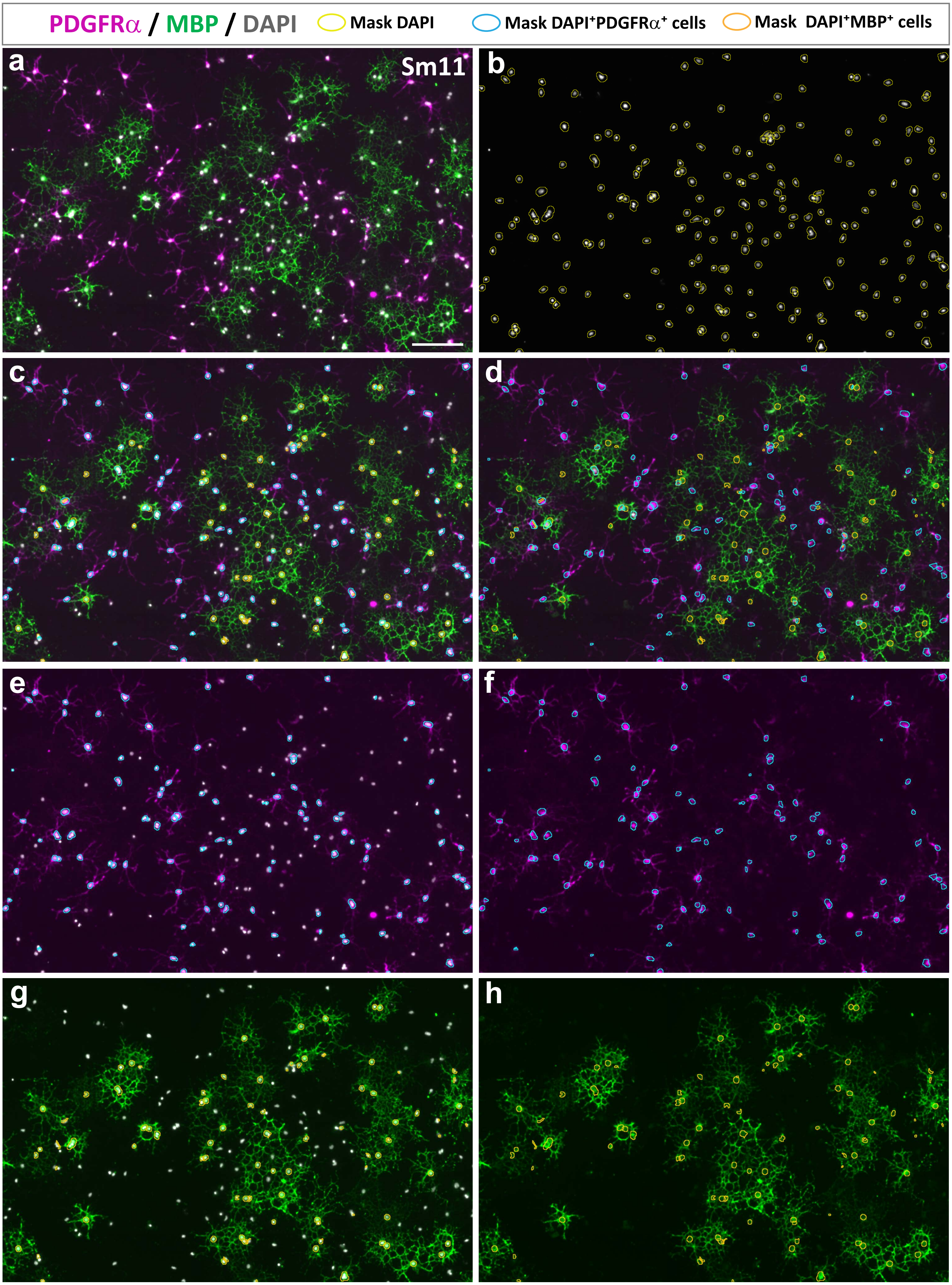
PDGFRα / MBP / DAPI
Mask DAPI
Mask DAPI+PDGFRα+ cells
Mask DAPI+MBP+ cells
a
Sm11
b
c
d
e
f
g
h

Supplementary figure 5. Automatic quantification of OPC differentiation cultures. (**a**) Representative image illustrating the immunodetection of OPCs (PDGFRα^+ cells, magenta) and differentiating OLs (MBP$^+$ cells, green) together with nuclear DAPI staining (grey) from OPC differentiation cultures, used to illustrate the automatic segmentation and quantification shown in the following panels. (**b**) Image illustrating with yellow masks the segmentation of DAPI nuclei (grey) used for automatic quantification. (**c**-**h**) Images illustrating with cyan masks the automatic identification of OPCs, DAPI nuclei having PDGFRα labeling (magenta), and with orange marks the identification of OLs, DAPI nuclei having MBP labeling (green), shown with (**c, e, g**) or without the DAPI channel (**d, f, h**), and showing PDGFRα and MBP labeling together (**c, d**) or as separate channels (**e-h**) to facilitate mask visualization. Scale bars: 20 μm.

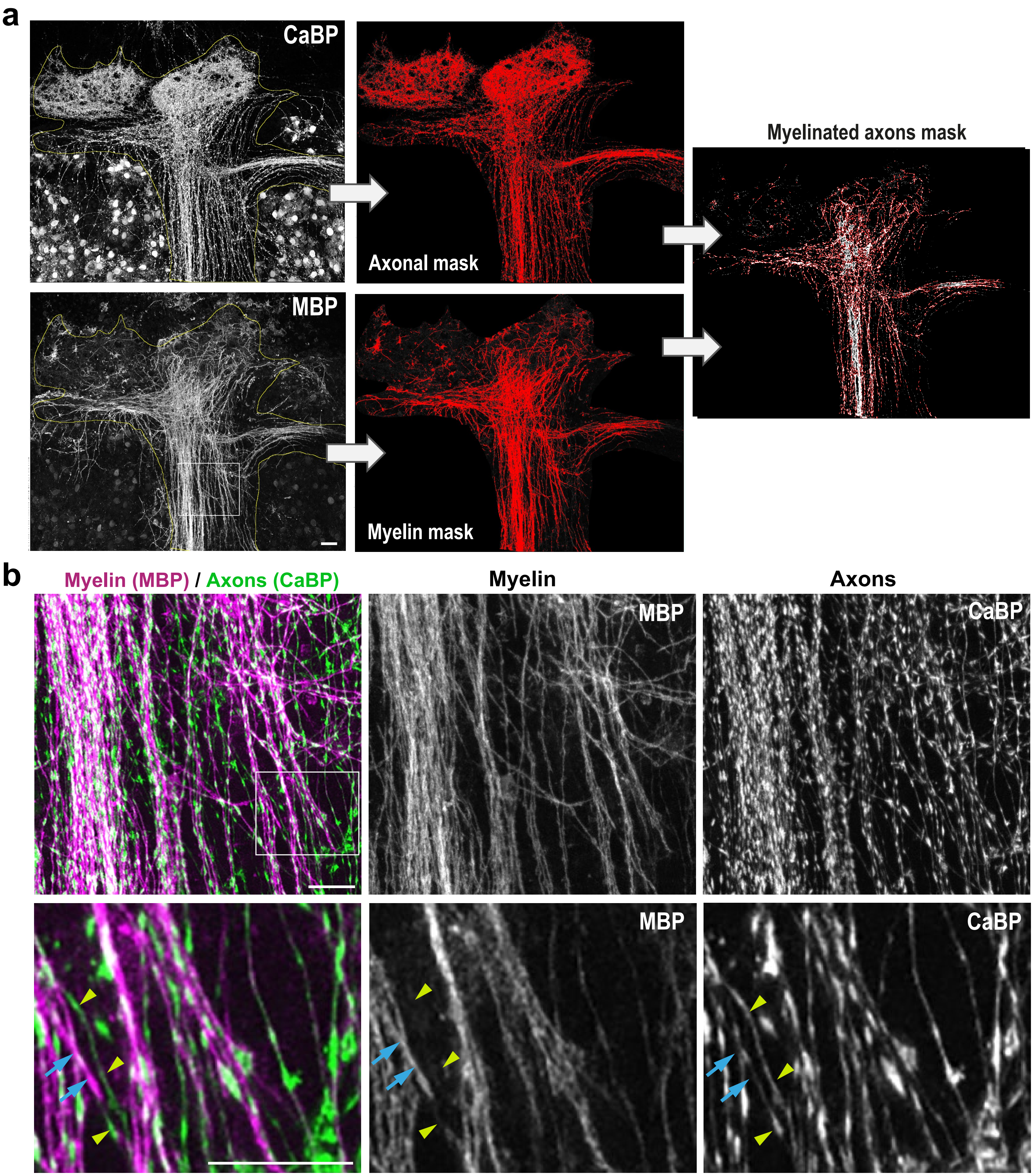

c

	Neonatal neural progenitors cultures						OPC cultures	Organotypic cerebellar slices		Rank
	iOLs diff	Oligodendroglia	Astrocyte	Neuron	Cell diff	Cell density	OL morphology	Differentiation	Myelination	
	SOX10$^+$	PDGFRα$^+$ CNP$^+$	GFAP$^+$	ß-III-tub$^+$	marker$^+$	DAPI	Cell surface (μm^2)	SOX10$^+$CC1$^+$/SOX10$^+$	CaBP$^+$/MBP$^+$	
Sm5	2.41	1.63	0.91	1.41	1.54	0.74	2.05	1.67	1.72	1
Sm11	2.24	1.47	0.86	1.65	1.43	1.01	2.04	1.30	1.84	2
Sm2	2.11	1.48	1.08	1.29	1.61	0.86	1.69	1.83	1.59	3
Sm1	2.04	1.50	0.93	1.15	1.35	0.87	1.83	1.59	1.47	4
T3	2.37	1.08	0.89	1.15	1.5	0.82	1.44	1.07	1.81	5
Sm7	1.62	1.46	1.05	1.14	1.36	0.95	1.37	1.48	1.12	6
Sm6	1.99	1.55	1.04	0.90	1.40	0.96	1.37	1.34	1.35	7
Clem	0.91	1.25	1.14	0.98	1.05	0.69	NA	1.67	1.26	8

Scale
>2
1.5-2
1-1.5
0.9-1.1
0.6-0.9

Supplementary figure 6. Automatic quantification of differentiation and myelination index in ex vivo cerebellar cultures, with a ranking of selected drugs for their pro-oligodendrogenic effects. (**a**) Illustration images of the automatic quantification approach to obtain the myelinating index (as optimized in Baudouin et al., 2021). (**b**) High magnification images illustrating the overlap between myelin, MBP immunodetection (magenta), and Purkinje neuronal axon immunodetected with CaBP antibody (green). Examples of myelinated- (blue arrows) and non-myelinated (yellow arrowheads) axonal segments are indicated in the lower panels. (**c**) Table integrating and ranking the effects of selected compounds on oligodendrogenesis. Biological effects of the small molecules and positive controls (T3, clemastine) determined through *in vitro* and *ex vivo* systems allow ranking them for their pro-oligodendrogenic activity to select the top two candidates for *in vivo* analysis. Data are presented as fold change compared to their respective vehicle. Diff, differentiation; NA, not assessed; Clem, clemastine. Scale bars: 20 µm in a panel, 10 µm in b panels.

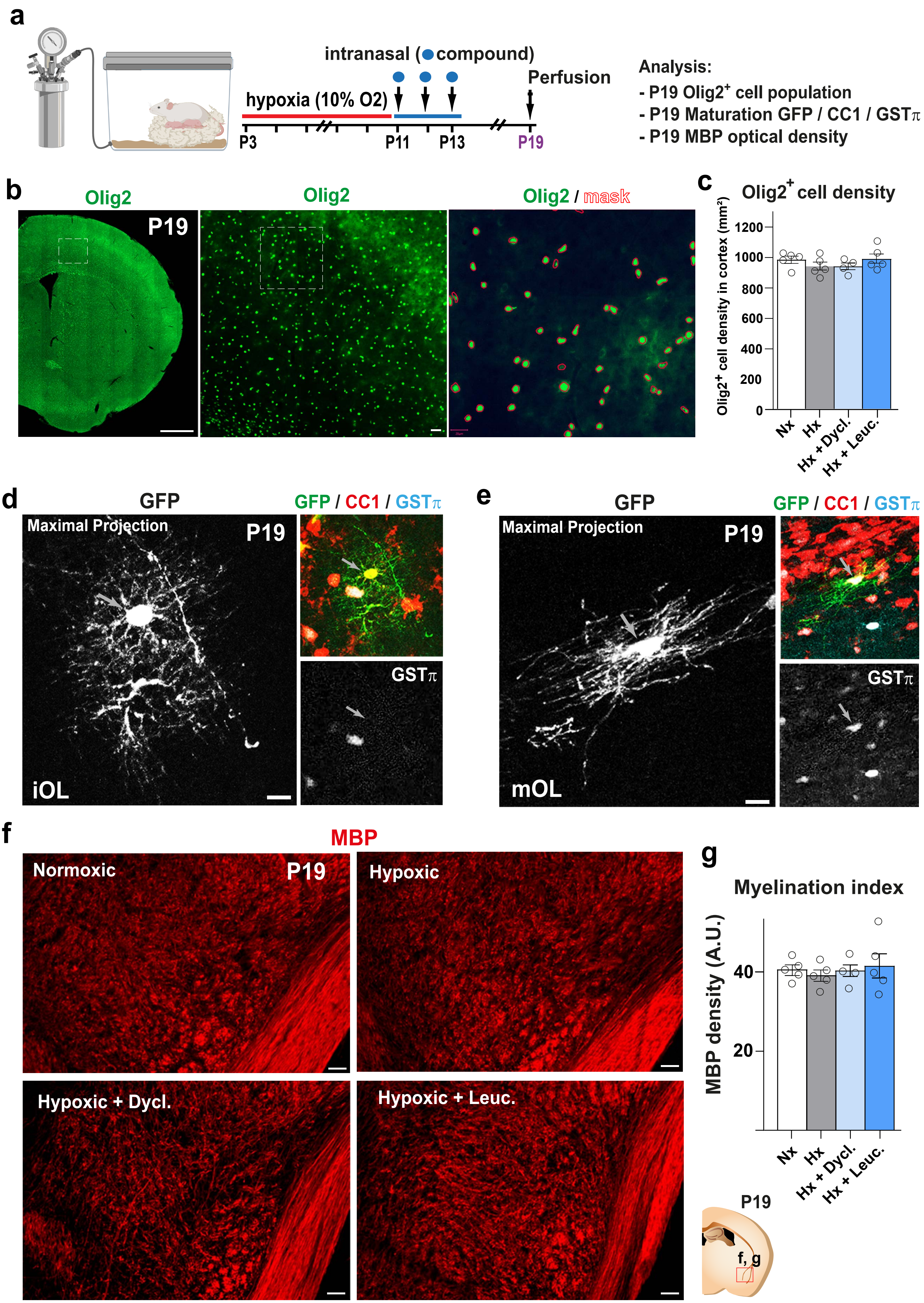

a
intranasal (● compound)
Perfusion
hypoxia (10% O2)
P3
P11
P13
P19
Analysis:
- P19 Olig2+ cell population
- P19 Maturation GFP / CC1 / GSTπ
- P19 MBP optical density
b
Olig2
P19
Olig2
Olig2 / mask
c
Olig2+ cell density
Olig2+ cell density in cortex (mm2)
1200
1000
800
600
400
200
0
Nx
Hx
Hx + Dycl.
Hx + Leuc.
d
GFP
GFP / CC1 / GSTπ
Maximal Projection
P19
GSTπ
iOL
e
GFP
GFP / CC1 / GSTπ
Maximal Projection
P19
GSTπ
mOL
f
MBP
Normoxic
P19
Hypoxic
Hypoxic + Dycl.
Hypoxic + Leuc.
g
Myelination index
MBP density (A.U.)
40
20
Nx
Hx
Hx + Dycl.
Hx + Leuc.
P19
f, g

Supplementary figure 7. Dyclonine and leucovorin promote oligodendroglial regeneration in a mouse model of preterm birth brain injury. (**a**) Schematic illustrating the workflow used to assess the capacity of dyclonine and leucovorin to promote OPC proliferation and rescue OLs maturation following neonatal chronic hypoxia. (**b, c**) Olig2 immunodetection and quantification showing that the density of OLs is not changed by hypoxia nor by treatment within the cortex at P19 (**c**). Quantification was performed via automated detections (QPath) as illustrated by the mask used for cellular segmentation in the third illustration (mask). (**d, e**) Illustrations validating the use of CC1 and GSTπ markers to identify OLs and mature/myelinating OLs respectively. Note that during OL differentiation, immature OLs (labeled with GFP) present a stellar morphology and are positive for CC1 but not for GSTπ (**d**), while mature/myelinating OLs having myelin segments co-express CC1 and GSTπ (**e**). (**f, g**) Representative images illustrating myelination by MBP immunodetection in the hypothalamic lateral zone, showing no difference between groups at P19 (**f**) and quantification of MBP immunofluorescence in this zone is represented in arbitrary units (A.U.) (**g**). Data are presented as Mean ± SEM. Statistics were performed using One-way ANOVA to compare the cell counts across different treatments followed by Dunnett's test to compare each treatment with either Normoxia (Nx) or Hypoxia considered as control groups. Each dot corresponds to a biological replicate. Exact p-values, sample sizes (represented in the dot plots), and source data are provided in the Source Data file and in Methods Table 3. Scale bars: 100 µm in b left panel, 20 µm in b right panels, and f; e; 4 µm in **d** and **e**.

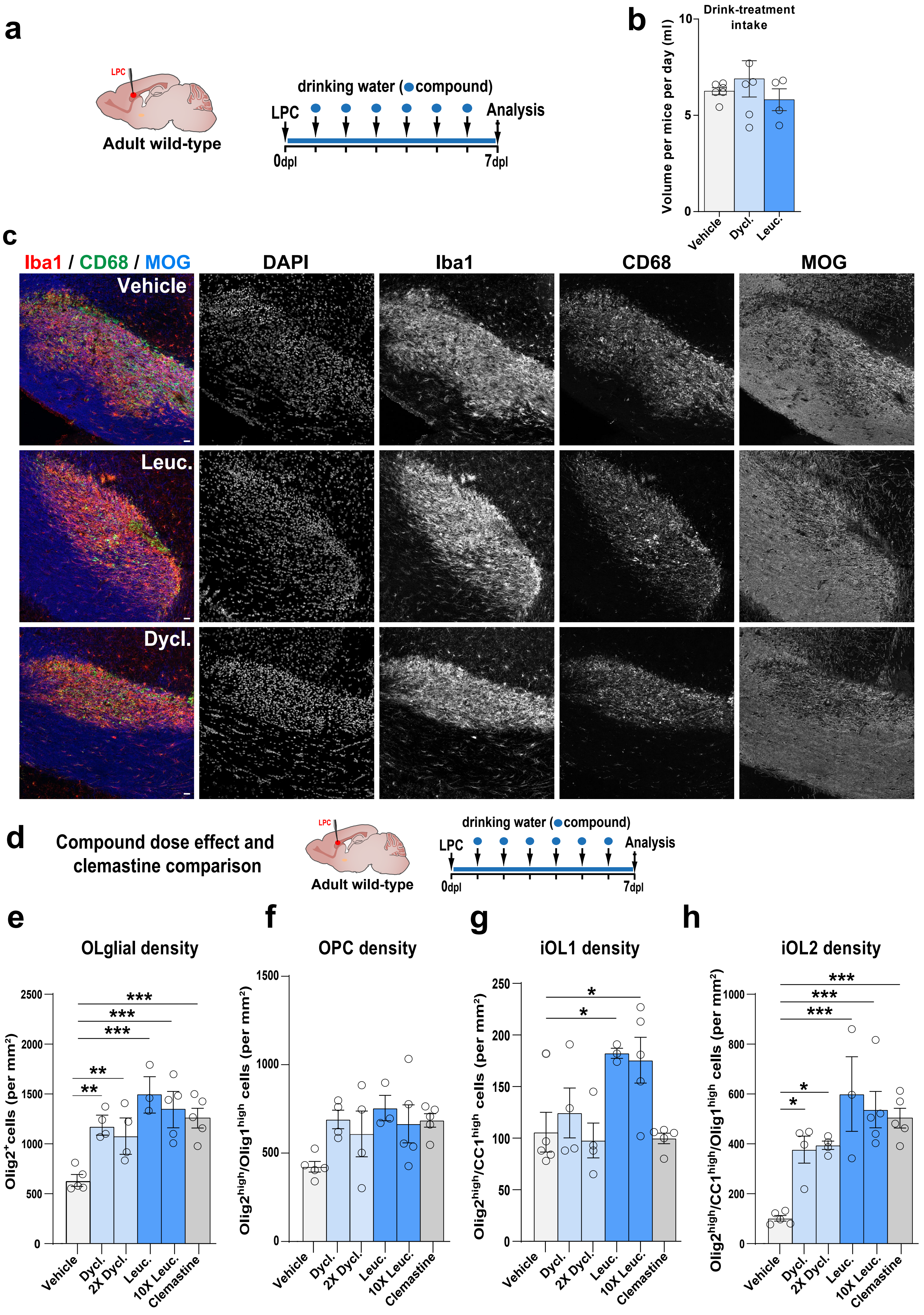

a
LPC
Adult wild-type
drinking water (●compound)
LPC
Analysis
0dpl
7dpl
b
Drink-treatment intake
Volume per mice per day (ml)
10
5
0
Vehicle
Dycl.
Leuc.
c
Iba1 / CD68 / MOG
DAPI
Iba1
CD68
MOG
Vehicle
Leuc.
Dycl.
d
Compound dose effect and clemastine comparison
LPC
Adult wild-type
drinking water (●compound)
LPC
Analysis
0dpl
7dpl
e
OLglial density
Olig2⁺cells (per mm²)
2500
2000
1500
1000
500
0
***
***
***
**
**
Vehicle
Dycl.
2X Dycl.
Leuc.
10X Leuc.
Clemastine
f
OPC density
Olig2^high/Olig1^high cells (per mm²)
1500
1000
500
0
Vehicle
Dycl.
2X Dycl.
Leuc.
10X Leuc.
Clemastine
g
iOL1 density
Olig2^high/CC1^high cells (per mm²)
250
200
150
100
50
0
*
*
Vehicle
Dycl.
2X Dycl.
Leuc.
10X Leuc.
Clemastine
h
iOL2 density
Olig2^high/CC1^high/Olig1^high cells (per mm²)
1000
800
600
400
200
0
***
***
***
*
*
Vehicle
Dycl.
2X Dycl.
Leuc.
10X Leuc.
Clemastine

Supplementary figure 8. Dyclonine and leucovorin promote both OPC proliferation and differentiation in a mouse model of adult demyelination. (**a**) Schematics illustrating the protocol for LPC demyelination in the corpus callosum, the timing of compound administration in drinking water and analysis. (**b**) Quantification of drinking intake per day (ml) during treatment showing no difference between groups and thus confirming the expected compound dose administration. (**c**) Single channel panels for the immunofluorescence in the lesion site identified by cell density (DAPI), microglia/macrophages (Iba1^{+} cells, red) and their phagocytic profile (CD68^{+} cells, green), and the reduction in myelin immunodetection (MOG, blue) in different treatment conditions. (**d**) Schematics illustrating the protocol of a replicated experiment to that of figure 7, comparing different doses of leucovorin and dyclonine, and using clemastine as a pro-oligodendrogenic positive control. (**e-h**). Quantification of Olig2^{+} oligodendroglial density (**e**), OPC density (**f**), iOL1 density (**g**) and iOL2 density (**h**) showing a similar increase at both doses tested of leucovorin and dyclonine in the density of oligodendroglia (Olig2+ cells), iOL1s (only increased by leucovorin treatment), and iOL2s. Note that clemastine induces a similar effect to dyclonine, with only leucovorin increasing the density of iOL1s. Dycl., dyclonine; Leuc., leucovorin; 2X Dycl. 2-fold dyclonine dose; 10X Leuc., 10-fold leucovorin dose; Clem., clemastine. Data are presented as Mean ± SEM. Each dot corresponds to a biological replicate. Statistics were performed using One-way ANOVA to compare the cell counts across different treatments followed by Dunnett's test to compare each treatment with Vehicle (control group). *p <0.05; **p <0.01; ***p < 0.001. Exact p-values, sample sizes (represented in the dot plots), and source data are provided in the Source Data file and in Methods Table 3. Scale bars: 20 mm.

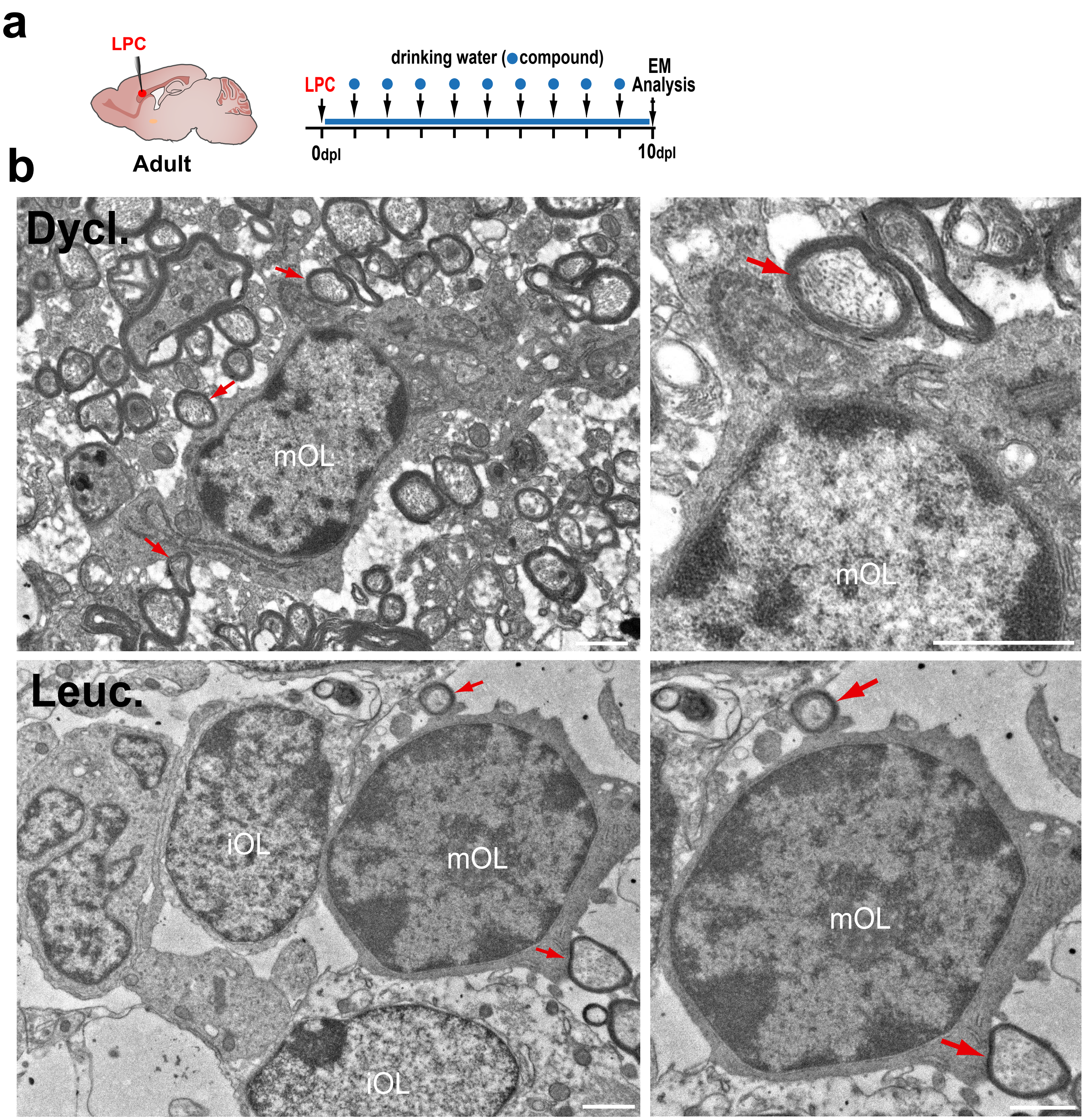
a
LPC
Adult
drinking water (● compound)
LPC
EM
Analysis
0dpl
10dpl
b
Dycl.
mOL
mOL
Leuc.
iOL
mOL
mOL
iOL

Supplementary figure 9. Leucovorin and dyclonine accelerate the generation of myelinating oligodendrocytes in a mouse model of adult demyelination. (a) Schematics illustrating the protocol used for compounds' administration in the drinking water and ultrastructural analysis by EM at 10 days post-lesion (dpl). **(b)** High magnification micrographs illustrating newly formed (re)myelinating OLs (mOL) in the lesion area, identified by their typical ultrastructural traits, i.e., oval-shape nucleus having densely packed chromatin with several heterochromatin spots and large cytoplasm in continuity with axons presenting compact myelin ultrastructure (red arrows) in dyclonine- and leucovorin-treated animals. Two examples of immature OLs (iOL), characterized by less compacted chromatin (lighter nuclear contrast than mOLs) and large cytoplasmic processes, are also shown in the bottom-left panel. The right panels show higher magnifications to better visualize the continuity between the OL cytoplasm and the myelinated axons (red arrows). Dycl., dyclonine; Leuc., leucovorin. Scale bars: 1 mm.

Reference BBB.

S8-1	Li, H. et al. Journal of Chemical Information and Modeling, 45(5), 1376-1384.
S8-2	Majumdar, S.et al. Molecular Informatics, 38(8-9), 1800164.
S8-3	Chico, L. K. et al. Nature reviews Drug discovery, 8(11), 892-909.
S8-4	Michelot J et al; Biopharm Drug Dispos 7: 197-206 (1986)
S8-5	Martins, I. F. et al. Journal of chemical information and modeling, 52(6), 1686-1697.
S8-6	Ghose, A. K. et al. ACS chemical neuroscience, 3(1), 50-68.
S8-7	Harvard Dataverse, https://dataverse.harvard.edu/dataset.xhtml?persistentId=doi:10.7910/DVN/21LKWG
S8-8	Kortagere, S. et al; Pharmaceutical research, 25, 1836–1845 (2008)25(8), 1836.
S8-9	Andres, C. et al. QSAR & Combinatorial Science, 25(4), 305-309.
S8-10	Gupta, M. et al; Journal of medicinal chemistry, 62(21), 9824-9836.
S8-11	https://www.boehringerone.com (Monography BERODUAL®)
S8-12	Shi M. J Neurotrauma. 2022 Sep;39(17-18):1240-1261.
S8-13	Madgula VL. Et al; Planta Med. 2010 Apr;76(6):599-606.
S8-14	Subramanian, G. et al. Journal of Computer-Aided Molecular Design, (2003), 17(10), 643-664.
S8-15	Mu E, Kulkarni J. et al. Aust Prescr. 2022 Jun;45(3):75-79.
S8-16	Zhang JX, et al ; Molecules. 2017 Feb 24;22(3):334.
S8-17	Adenot, M. et al. Journal of chemical information and computer sciences, 2004, 44(1), 239-248.
S8-18	Tan DX. Et al. Curr Neuropharmacol. 2010 Sep;8(3):161.
S8-19	Youdim KA et al. Free Radic Biol Med. 2004 Mar 1;36(5):592-604.
S8-20	Drugbanks : https://www.drugbank.ca/drugs/DB11632
S8-21	Shelmire B. *AMA Arch Derm.* 1955;71(6):728–730.
S8-22	Schwarz T. et al. Chem Med Chem 2016,11,1380–1394
S8-23	Mochizuki T et al. Brain Res. 1996 Dec 16;743(1-2):178-83.
S8-24	Ishizaki J et al; Biol Pharm Bull 21 (1): 67-71(1998)
S8-25	Parry GJ et al. Clin Neuropharmacol. 2010 Jan-Feb;33(1):17-21.
S8-26	Schutta H.S. et al. The Journal of Pediatrics. Vol. 75, No. 6, part 1, pp. 1070-1079
S8-27	The European Agency for the Evaluation of Medicinal Products. EMEA/MRL/256/97-FINAL. October 1997
S8-28	Quinn M. et al. Dig Liver Dis. 2014 Jun; 46(6):527-34.
