# Supplementary material for: Pharmacogenomic screening identifies and repurposes leucovorin and dyclonine as pro-oligodendrogenic compounds in brain repair
Source: Nat Commun. 2024 Nov 13;15:9837. doi: 10.1038/s41467-024-54003-9 (PMC11561360; doi:10.1038/s41467-024-54003-9)
Supplement: Supplementary file 2 — Description of Additional Supplementary Files [file 41467_2024_54003_MOESM2_ESM.docx]

**Description of Additional Supplementary Files**

**Supplementary Data 1 Supplemental tables 1 to 12**

1. Table 1: Broad transcriptional signature of oligodendroglial lineage cells
2. Table 2: Transcriptional signature of genes expressed in dorsal and lateral neonatal subventricular progenitors (NSCs & TAPs)
3. Table 3: Oligodendroglial genes enriched in dorsal and lateral progenitors (NSCs & TAPs), or not
4. Table 4: Refined subset of genes involved in oligodendroglial development (3372 genes), GO analysis
5. Table 5: Correlated “hub genes” from SPIED analysis (632 genes), oligodendroglial genes, GO analysis
6. Table 6: Anti correlated “hub genes” from SPIED analysis (623 genes), GO analysis
7. Table 7: Pharmacogenomic scoring for drug selection (156 drugs)
8. Table 8: Pharmacological scoring for drug selection (40 drugs)
9. Table 9: Transcriptional hub genes regulated by Sm5 (Dyclonine) and GO associated
10. Table 10: Transcriptional hub genes regulated by Sm11 (Leucovorin) and GO associated
11. Table 11: OligoScore analysis of Sm5-regulated hub genes
12. Table 12: OligoScore analysis of Sm11-regulated hub genes

**Supplementary Data 2 Method tables 1 to 5**

1. Methods table 1: Names and characteristics of small molecules (sm) 1 to 11 and positive controls
2. Methods table 2: Main methodology and selection criteria during the bio-informatics screening
3. Methods table 3: Summary statistics
4. Methods table 4: List of primary antibodies
5. Methods table 5: List of secondary antibodies

**Supplementary Data 3 OligoScore validation tables**

Datasets related validation of OligoScore related to supplemental figure 3

**Supplementary Data 4 OligoScore hub genes Sm5.**

OligoScore output document upon querying with hub genes of Sm5

**Supplementary Data 5 OligoScore hub genes Sm11**

OligoScore output document upon querying with hub genes of Sm11
